# How life stressors influence modifiable lifestyle factors, depressive symptoms, and physical and mental health among Vietnamese older women?

**DOI:** 10.1186/s12888-017-1395-y

**Published:** 2017-06-29

**Authors:** Tiet-Hanh Dao-Tran, Debra Anderson, Charrlotte Seib

**Affiliations:** 1 Faculty of Nursing and Medical Technology, University of Medicine and Pharmacy, HCMC, Vietnam; 2 Center of Work, Organisation, and Well-being, Griffith University, Room 108, Building: N63, Nathan Campus, Brisbane, QLD4111 Australia; 3 School of Nursing, Queensland University of Technology, Brisbane, Australia

**Keywords:** Life stressors, Modifiable lifestyle factors, Depressive symptoms, Physical health, Mental health

## Abstract

**Background:**

Research has demonstrated that exposure to life stressors can influence health through a number of pathways. However, knowledge about the patterns of life stressors and their contributions to health in different populations is limited. Vietnamese older women have attracted little research to date in this area.

**Methods:**

This cross-sectional study used an interview-administered-questionnaire to collect data from 440 Vietnamese older women. Descriptive analysis was used to describe life stressors among Vietnamese older women. Binary analysis and Structural Equation Modelling statistical analysis were used to examine the influences of life stressors on modifiable lifestyle factors, depressive symptoms, physical and mental health among Vietnamese older women.

**Results:**

Vietnamese older women in this study commonly reported the experience of losing a close person, including a baby/child, serious health or money problems, violence and disaster. Among the study participants, (1) exposure to more life stressors increased their depressive symptoms, and decreased their physical and mental health; (2) exposure to more life stressors also increased their physical health by increasing their physical activity levels.

**Conclusion:**

Life stressors influenced health among Vietnamese older women through different pathways. Interventions to manage stress and depressive symptoms are required for Vietnamese older women in the future.

## Background

Research has demonstrated that exposure to life stressors does not only directly influence health [1], but can also influence health through three main pathways [2]. First, exposure to life stressors is linked to illness by changes in physiology, including the immune and cardiovascular systems, and endocrine reactivity, which causes illness [3]. Second, people who experience life stressors are more likely to adopt an unhealthy lifestyle [4–6], such as poor diet [7], smoking [4, 7], excess alcohol drinking [4, 5, 8], physical inactivity [4, 7], sleep disturbance [9, 10], and excess BMI [11], and these lifestyles are associated with decreased health [12–20]. Third, exposure to life stressors is linked with depression [21], and is associated with illness [22, 23]. However, the patterns by which life stressors influence health have been investigated in limited contexts. The majority of studies on life stressors and health have been done in Western cultures. Knowledge about life stressors and health in Asian cultures is limited.

Research has shown that compared to men, women usually have greater exposure to life stressors [24]. Also, even though exposed to the same life stressor, women also appear to be more vulnerable to the negative impacts of stress than men [5]. These experiences are particularly true for the women who are widowed, members of minority ethnic groups, or poorly educated [25]. Moreover, the number of people aged 60 and over has been increasing all over the world and is projected to comprise approximately 20% of the global population by 2050 [26]. Also, despite the innovations in health, aging is still linked to decreased health [27]. Among older populations, women comprise a higher proportion [26], and more frequently report lower levels of health than men [28–30]. Yet, only a small number of studies about life stressors and health have been conducted from gender or aging perspectives. Therefore, more research about life stressors and health among older women, especially these who experience disadvantage, is needed.

Currently, there are approximately five million older women in Vietnam [31]. Vietnamese older women have lived through wars and life after wars with limited resources [32]. Due to traditional gender beliefs, Vietnamese older women were generally less likely to receive education than men, and so had less chance for well-paid employment [33]. After marriage, the majority of Vietnamese older women have lived with their husbands’ families, where they are the main caretakers for the housework and the extended family members [34]. Recently, along with dramatic socio-economic changes, domestic violence has increased in Vietnam [35], with 58% of women in Vietnam suffering at least one kind of domestic violence in their lifetime [36, 37]. These factors suggest that Vietnamese older women may have experienced various life stressors, and their experience of life stressors may have influenced their health. Yet, to date, knowledge about life stressors and their impact on health among Vietnamese older women is largely unknown.

Furthermore, the majority of previous studies have used binary analysis, simple regression analysis or multiple regression analysis for data analysis. These data analysis approaches have limited investigation to direct influences among variables, and missed the opportunity to explore the indirect impact of stressors. A combination of the influences among variables found in different studies or data analysis in a comprehensive model might increase statistical errors. While structural equation modelling can increase statistical robustness in modelling a comprehensive model of both direct and indirect influences among variables, few studies have used this statistical approach for their data analysis. Therefore, the use of structural equation modelling to comprehensively model the impact of life stressors on modifiable lifestyle factors, depressive symptoms, physical and mental health among Vietnamese older women is needed.

This study aimed to describe the life stressors of Vietnamese older women, and use structural equation modelling to examine the influences of life stressors on modifiable lifestyle factors, depressive symptoms, and health among Vietnamese older women. As almost all of Vietnamese women abstain from smoking [38] and alcohol [39], the modifiable lifestyle factors in this study included vegetable and fruit consumption, physical activity levels, sleeping, and BMI. It is hoped that knowledge gained from this study will be useful for proposing interventions to promote health among Vietnamese older women in the future.

## Methods

### Study design, setting, and participants

This study used a cross-sectional design. This design is not ideal to confirm causal relationships among variables; however, this design is still a very useful approach to explore the relationships among study variables as long as there is a strong theoretical approach used to underpin the model’s specification. Also, while there was a constraint on resources, this design increased the study’ feasibility.

The study collected data from a random sample of 440 women, who were selected from contact lists provided by the Community Leaders of Older People’s Unions in 16 rural and urban suburbs in Vietnam from 8/2014–1/2015. Women were invited to participate if they were: (1) Vietnamese, (2) aged 60 years old and above, (3) able to communicate in Vietnamese, and (4), able to give informed consent.

### Hypothesis

In this study, it is preliminarily hypothesized that the number of life stressors would directly influence health [1]. It is also preliminarily hypothesized that the number of life stressors would also initiate or reinforce unhealthy diet [7], sedentary lifestyle [4, 7], sleep disturbances [9, 10], and elevated BMI [11]. With these health-compromising behaviours, participants may experience worse general physical and mental health [12, 13, 17, 19]. In addition, the study hypothesised that people exposed to more life stressors would be more likely to develop depressive symptoms [13, 40], and this could lead to decreased physical and mental health [22, 23].

### Measures

In this study, an anonymous structured validated questionnaire was used to collect data. The questionnaire included: the Life Stressors Checklist-revised (LSC-r) [41] to collect data about life stressors, standard questions to collect data about the average amount of vegetables, and fruits consumed everyday currently and current physical activity levels; the General Sleep Disturbance Scale [42] to measure sleep disturbances, the Centre for Epidemiologic Studies Depression (CES-D) Scale [43] to assess depression, and The Short Form 12 (SF12) [44] to collect data about physical and mental health. Due to the variation of the participants’ education levels, and their vision capacities, data were collected with an interview-administered-questionnaire. The participants were also measured for their weight and height by the researchers, from which BMI was calculated.

The Life Stressors Checklist-revised (LSC-r) [41] consists of 29 self-reported items about lifetime trauma exposures which are important in women’s entire lives, such as sexual or physical assault, losing a baby/child, or death of a person close to them. The scale is calculated by adding one point for each positively endorsed stressor. This allows the score for LSC-r to range from zero to 29, with higher LSC-R scores indicating the greater number of SLEs that the individual has experienced [41].

The General Sleep Disturbance Scale [42] consists of 21 items about sleep problems. Participants self-reported number of days they had experienced a sleep disturbance in the last seven days. The answers were rated from 0 (never) to seven (everyday). Total possible scores for this scale ranged from 0 to 147, and a total score of 43 or above represented sleep disturbance [42].

The Centre for Epidemiologic Studies Depression (CES-D) Scale [43] has 20 items. The instrument measures the frequency of experiencing depressive symptoms in the last 7 days. Participants described how many days they felt or behaved in the identified ways in the last 7 days. The answers are rated from 0 (less than 1 day) to 3 (5–7 days). The possible range of scores is zero to 60, with scores between 16 and 26 suggesting mild depression, and scores greater than 27 suggesting major depression [45].

The Short Form 12 (SF-12) [44] includes 12 questions about health. Participants are asked about their health during the past four weeks. The SF-12 is scored using a standard scoring procedure. The possible total scores for the Physical Component Summary (PCS) and the Mental Component Summary (MCS) range from 0 to 100. A higher score indicates a better health.

### Data analysis

Analysis was performed using SPSS 22.0 (Statistical Package for Social science Software), and AMOS 22.0 (Analysis of Moment Structures) [46]. As there was little random missing data, data were imputed using the expectation maximization functions in SPSS before the analysis [47]. Descriptive data were presented as counts, percentages, median, and range, as data were not normally distributed. Prior to testing the goodness of fit between the preliminary hypothesized model and the data, bivariate correlations among study variables were performed to finalize the hypothesized model. The Spearman rho tests were used to examine the association between two continuous variables as they were not normally distributed. To test the goodness of fit, structural equation modelling (SEM) statistical analysis was used [48]. The model was considered an adequate fit if it had: (1) a non-significant χ2 test; (2) 1 < χ^2^/*df* < 2 and; (3) a root mean square error of approximation (RMSEA) <0.05 and p of Close Fit (PCLOSE) > 0.05; (4) an adjusted goodness of fit index (AGFI) > 0.9; and (5), a standardized root mean square residual (SRMR) <0.06 [48]. Prior to performing SEM, the assumptions of normal distribution and outliers were examined [48]. As data were not normally distributed, Structural Equation Modelling using the Asymptotic Distribution Free estimator (ADF) and the Bollen-Stine bootstrap p function was applied to test the goodness of fit between the final hypothesized model and the study data. The level of significance was set at α = 0.05 [49].

## Results

### Description of the sample

Table 1 presents descriptions of the socio-demographic characteristics of the sample. Overall, 440 women aged between 60 and 94 years (median = 68) participated in the study. The majority of the sample had completed primary school or less (75.9%), and were not currently employed (70.2%). Around half of the women lived with their partners or husbands (50.2%) and had low income (53.0%).

**Table 1:** Socio-demographic characteristics of the study sample (*n* = 440)

Variable	*N* (%)
Age (median (range)	68 (60–94)
Living areas	
Rural	312 (70.9)
Urban	128 (29.1)
Marital status	
Single (never married)	11 (2.5)
Married/ partnered	221 (50.2)
Widow/divorced/separated	208 (47.3)
Highest educational achievement	
Primary or less	334 (75.9)
Junior school	59 (13.4)
Senior school	31 (7.0)
Diploma, university	16 (3.6)
Currently employed	131 (29.8)
Average month income*	
Low (about <80 USD)	233 (53.0)
Middle (about 80- <450 USD)	189 (43.2)
Rather high (about 450 - <1000 USD)	12 (2.7)
High (about ≥1000 USD)	2 (0.5)

* Income categories derived from the national tax payment standard

### Life stressors among Vietnamese older women

Table 2 presents life stressors among Vietnamese older women. Overall, the older Vietnamese women reported a range of 0–22 life stressors out of 29 (median = 4). The most commonly reported life stressors among the sample included losing a loved one (93.6%), abortion or miscarriage (48.9%), serious money problems (42.3%), serious physical or mental illness (35.2%), or experiencing a natural disaster (31.6%). Several Vietnamese older women also reported experiences of some form of violence (either physical, emotional or sexual) (3.0%; 13.4%; 10.9% respectively), caring for a handicapped child or close people with some severe health problems (24.3%), or having close family members or herself serve time in jail (13% or 3%).

**Table 2:** Life stressors reported by Vietnamese older women (*n* = 440)

		*n* (%)
1	Serious disaster	139 (31.6)
2	Witnessed serious accident	59 (13.4)
3	Experienced serious accident	70 (15.9)
4	Close family member in jail	44 (10.0)
5	Sent to jail	13 (3.0)
6	Been put in foster care or put up for adoption	24 (5.5)
7	Parents separated or divorced while living with them	10 (2.3)
8	Separated or divorced	38 (8.6)
9	Serious money problems	186 (42.3)
10	Serious physical or mental illness	155 (35.2)
11	Emotionally abused or neglected	60 (13.6)
12	Physically neglected	13(3.0)
13	Experienced abortion or miscarriage (lost your baby)	215 (48.9)
14	Separated from your child against your will	12 (2.7)
15	Your child with a severe physical or mental handicap	25 (5.7)
16	Caring for close one with a severe physical or mental handicap	82 (18.6)
17	Close one died suddenly or unexpectedly	125 (28.4)
18	Someone close to you died (not suddenly)	412 (93.6)
19	Witnessed violence between family members (before age 16)	12 (2.7)
20	Seen a robbery, mugging, or attack taking place	31 (7.0)
21	Been robbed, mugged, or physically attacked by strangers	33 (7.5)
22	Physically attacked by someone you knew (before age 16)	3 (0.7)
23	Physically attacked by someone you knew after 16	56 (12.7)
24	Bothered or harassed by sexual remarks, demands at work/school	4 (0.9)
25	Forced to be touched or touch someone in a sexual way (before 16)	2 (0.5)
26	Forced to be touched or touch someone in a sexual way after 16	5 (1.1)
27	Forced to have sex (before age 16)	1 (0.2)
28	Forced to have sex after 16	36 (8.2)
29	Other event experienced by someone close to you	41 (9.3)

### The influences of life stressors on modifiable lifestyle factors, depressive symptoms, and physical and mental health among Vietnamese older women

**Fig. 1:**
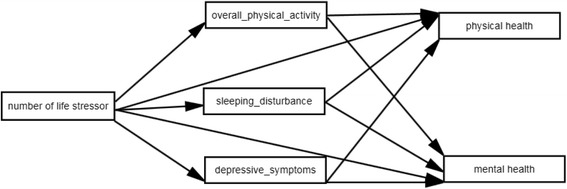
Final hypothesized model about the influences of life stressors

Table 3 presents the correlation matrix between observed continuous variables. The table indicates that there were significant correlations between life stressors reported and overall physical activity levels, sleep disturbance, depressive symptoms, physical health, and mental health. Among the lifestyle factors and depressive symptoms, several correlations existed. The table also shows significant correlations between vegetable consumption and physical health; fruit consumption and physical and mental health; overall physical activity levels and physical health; depressive symptoms and physical and mental health. Based on these findings, the preliminary hypothesis was modified and the study hypothesized model was finalized. This model was tested for the goodness of fit between the hypothesized model and the study data (Fig. 1).

**Table 3:** Spearman rho correlation matrix among the study variables

	1.	2.	3.	4.	5.	6.	7.	8.	9.
1. Number of SLEs	1								
2. Vegetable serves/day	.012	1							
3. Fruit serves/day	−.057	.440^**^	1						
4. Overall physical activity level	.135^**^	.157^**^	.024	1					
5. Sleep disturbance	.126^**^	−.184^**^	−.165^**^	−.144^**^	1				
6. BMI	.003	.123^**^	.102^*^	.041	−.067	1			
7. Depressive symptoms	.380^**^	−.244^**^	−.222^**^	−.050	.519^**^	−.098^*^	1		
8. Physical health	−.131^**^	.267^**^	.199^**^	.340^**^	−.435^**^	.024	−.366^**^	1	
9. Mental health	−.241^**^	.096^*^	.208^**^	−.059	−.269^**^	.071	−.372^**^	.001	1

^*^ *p* < 0.05 ^**^ *p* < 0.01

**Fig. 2:**
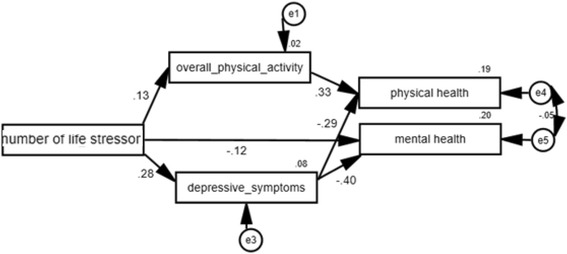
Final model about the influences of life stressors with standardized estimates

Because the hypothesized model did not fit the study’s data, modification was performed. Figure 2 displays the standardized path coefficients of the final model. All paths were significant at *p* < 0.05. The model was fit with (1) *p* = 0.31; (2) χ2/df = 1.19; (3) RMSEA = 0.02; PCLOSE =0.69; GFI > 0.99; Adjusted GFI = 0.99; SRMR = 0.02. The modified model indicated that among Vietnamese older women, the number of life stressors directly influenced mental health (β = −0.12, *p* < 0.05). The model also showed that the number of life stressors experienced indirectly influenced physical health (β = −0.29, *p* < 0.05) and mental health (β = −0.40, *p* < 0.05) through depression level (β =0.28, *p* < 0.05). In addition, the number of life stressors indirectly influenced physical health (β =0.33, *p* < 0.05) through physical activity levels (β = 0.13, *p* < 0.05).

## Discussion

This study explored life stressors, and examined the influences of life stressors on modifiable lifestyle factors, depressive symptoms, and physical and mental health among Vietnamese older women. In regard to life stressors, the study found that many Vietnamese older women experienced caring for those with serious health problems and violence, and domestic violence. Vietnamese girls did not receive equivalent education to boys and so frequently had fewer opportunities for paid employment [33]. Growing up, they mainly took care of housework, and sick family members [34]. Many Vietnamese older women had been taught to “obey their father when they are young, obey their husbands when they get married and obey their children when their husbands passed away”. Consequently, they might have believed that their husbands had the right to order them what to do, and husbands could have employed punishment on their wives. The fact is that many Vietnamese women reported domestic violence [36, 37]. Yet, the social norms that existed in Vietnam have inhibited women from marital changes [37]. This may have increased the risks of suffering from domestic violence.

The study also found that a number of Vietnamese older women experienced family members or themselves in jail, losing a baby/child, and serious physical or mental illness or money problems. There are several possible explanations for this finding. Vietnamese older women have lived through wars against the French, Japanese, Americans, the Pol Pot government, and China. During the wars, people were more likely to experience being in jail or having family member(s) in jail. Until the 1990s, many Vietnamese people still lived with very limited social, economic, and medical resources [32]. Consequently, the abortion rate in Vietnam rose rapidly between 1976 and 1987, from 70,281 cases to 811,176 cases [50]. Many children died because of accidents [51, 52]. Two thirds of children aged six and less in low income families did not receive treatment or they treated themselves when they were sick [52]. These factors may account for women experiencing health or financial problems and the loss of a baby/child. However, about two thirds of the data were collected from Dong Thap province, where the majority of residents lived on agriculture. In 1978, this area experienced a serious flood, and as a result, many people living in this area experienced famine and poverty. In this context, the prevalence of experience of disaster and serious money problems could be over-reported and so interpretation of these data for the general Vietnamese older women’s population should be undertaken with caution.

Concerning the influences of life stressors on modifiable lifestyle factors, depressive symptoms, and health among Vietnamese older women, the study’s findings are consistent with contemporary literature and support the prevailing theory to a certain extent. More specifically, the study indicated that life stressors directly impacted on mental health [53, 54]. In addition, the study also indicated that life stressors were linked with depression [5, 22, 55], which can in turn contribute to decreasing both mental and physical health [23]. Previous studies also indicated that after adjusting for marital status, education, income, employment status, pregnancy status, or being abused as a child, those who experienced abuse in their adult intimate relationships would be more likely to experience depression than others [56] and, in turn, depression significantly contributed to decreasing health [55]. Taking both the direct and indirect impacts of life stressors on health together, the study’s findings suggest that life stressors had moderate impacts on depressive symptoms, and physical, and mental health among Vietnamese older women.

However, two findings from this study are not congruent with the theory and findings from other populations [57]. One of the differences is the associations between exposure to life stressors and diet, sleep, problems and BMI. Previous studies suggested that exposure to life stressors was linked with an unhealthy diet [7], sleep disturbance [9, 10], and excess BMI [11]. Then, these lifestyles are associated with decreased health [12–20]. These associations were not found among Vietnamese older women. A possible explanation is that Vietnamese older women may have experienced the listed life stressors a long time ago, and the impact of this experience may have decreased over time and may no longer have an influence on their current diet, sleep and BMI. Another difference is in the associations between exposure to life stressors and physical activity, and health. Schwarzer & Schulz [58] posited that those exposed to stressors would be more likely to have a sedentary lifestyle [59], and as the result, people would experience decreased physical and mental health [60]. Among Vietnamese older women, those who reported more life stressors also reported more overall physical activity and these increased physical activity levels then increased their physical health. There are a number of possible explanations for this difference. One possible explanation is that older women in Vietnam may believe that the most common source of their life stressors comes from their low socio-economic status. They may have tried to work harder rather than being sedentary to overcome this situation. Another possible explanation is that Vietnamese older women may have used avoiding stress coping strategies to deal with their stressors. They may have increased physical activity to avoid thinking too much about their life stressors. However, as these possible explanations have not been studied in previous studies, an in-depth interview to further investigate how Vietnamese older women appraise their life stressors and their common coping strategies would be useful to provide better explanations for these interesting findings among Vietnamese older women.

### Strengths and limitations

This study has several strengths. First, the study was conducted in a previously unstudied population and provided knowledge of life stressors and their sequelae among Vietnamese older women for the first time. Secondly, as the participants were recruited through the Older People’s Unions in the communities, the study captured a significant proportion of older women in the research areas. Thirdly, the data for this study were collected through Vietnamese versions of validated instruments, which achieved good translation equivalence and internal consistency. As such, the instruments improved the reliability of the study’s findings and provide a basis for further data collection. Fourthly, as the study used SEM for data analysis, both direct and indirect influences of life stressors on modifiable lifestyle factors, depressive symptoms, physical health, and mental health among older women were explored.

However, the study also had a number of limitations. First, this study used face-to-face interviews to collect data which might have introduced social bias, and reporting bias. To minimise this, interviews were conducted in a private location to limit the participants from over or under reporting. Second, as the study asked participants about their life time stressors, recall bias may have occurred. Third, the participants experienced wars, but the LSC-R which was used to measure life stressors does not account for wars. Fourth, since this is a cross-sectional study, findings about relationships among study variables could not be concluded as causal. Fifth, while research suggested that more recent exposure to stressors could have a stronger impact on lifestyle and health, this study did not compare the impact of life stressors by their exposure time. Sixth, perceived stress, rather than the stressor itself, can have a stronger influence on health [61], and the study did not include perceived stress in the model. Finally, while exposure to stressors has been linked to illness by changing physiology [3, 62, 63], this study did not include immune, cardiovascular, and endocrine parameters due to limited time and facilities.

## Conclusions

This study found that Vietnamese older women experienced various life stressors and exposure to life stressors influenced modifiable lifestyle factors, depressive symptoms, and that health among Vietnamese older women differently. Similar to other cultures, exposure to more life stressors increased depressive symptoms, and decreased physical and mental health among older women in Vietnam. However, among older women in Vietnam, exposure to more life stressors was not linked to diet, sleep problems, and BMI. In fact, it increased their physical health by increasing physical activity levels.

At the moment, the health care system in Vietnam mostly focuses on physical diseases, and available resources and facilities for mental health care in this context are still limited. Findings from this study suggest that further service provisions to promote mental health are required for Vietnamese older women. A development of primary mental healthcare services and psychological counselling services for stress and depressive symptom management might be a helpful approach to promote health for Vietnamese older women in the future.

## Abbreviations

ADF: Asymptotic Distribution Free estimator
AGFI: An adjusted goodness of fit index
AMOS: Analysis of Moment Structures
BMI: Body Mass Index
CES-D: The Centre of Epidemiologic Studies Depression Scale
GSDS: General Sleep Disturbance Scale
LSC-r: Life Stressors Checklist – revised
MCS: Mental health component scores
PCLOSE: p of Close Fit
PCS: Physical health component scores
RMSEA: A root mean square error of approximation
SEM: Structural equation modelling
SF12: The Short-Form 12 items
SLEs: Stressful life events
SPSS: Statistical Package for Social Sciences
SRMR: A standardized root mean square residual
